# Real-life Progression of the Use of a Genetic Panel in to Diagnose Neonatal Cholestasis

**DOI:** 10.1097/PG9.0000000000000196

**Published:** 2022-03-31

**Authors:** Shogo Ito, Takao Togawa, Kazuo Imagawa, Koichi Ito, Takeshi Endo, Tokio Sugiura, Shinji Saitoh

**Affiliations:** From the * Department of Pediatrics and Neonatology, Nagoya City University Graduate School of Medical Sciences, Nagoya, Japan; † Department of Child Health, Faculty of Medicine, University of Tsukuba, Ibaraki, Japan; ‡ Department of Pediatrics, Nagoya City University East Medical Center, Nagoya, Japan.

**Keywords:** neonatal intrahepatic cholestasis, next-generation sequencing, molecular genetic diagnosis, genetic liver diseases, Crigler-Najjar syndrome

## Abstract

The study aimed to construct an advanced gene panel to ascertain the genetic etiology of patients with neonatal/infantile intrahepatic cholestasis (NIIC), and test patients with NIIC in a clinical setting.

**Methods:**

From the group of NIIC patients, whom we had previously tested with our old 18-gene panel from May 2013 to September 2017 but could not establish a definitive diagnosis, we included 191 in the retrospective reanalysis group for this study. Additionally, we recruited 124 patients with NIIC into a prospective analysis group from October 2017 to October 2019. Cholestasis was defined as a serum direct bilirubin level >1.0 mg/dL. We constructed a 61-gene panel for targeted next-generation sequencing of the patients.

**Results:**

In the retrospective reanalysis group, we found mutations in *ABCC2*, *MPV17*, *NPC1*, *CFTR*, *NR1H4*, or *CYP27A1* in 10 (5.2%) of the 191 patients. In the prospective analysis group, 33 (26.6%) of the 124 patients had a causative mutation in *JAG1*, *NOTCH2*, *ABCC2*, *SLC25A13*, *ABCB11*, *POLG*, *NPC1*, *CFTR*, *ATP8B1*, or *ABCB4*. The top 3 genetic diagnoses were of Alagille syndrome, neonatal Dubin-Johnson syndrome, and neonatal intrahepatic cholestasis caused by citrin deficiency, which together constitute 78.8% of the genetic causes of cholestasis in Japan. We also identified 3 genotypes associated with Crigler-Najjar syndrome type 2 in the retrospective reanalysis group.

**Conclusions:**

The advanced NIIC gene panel successfully uncovered molecular genetic etiologies of NIIC not only in the reanalysis group but also in the prospective cohort. Crigler-Najjar syndrome type 2 patients may be included along with NIIC patients.

**What is Known**

- Next-generation sequencing has helped in establishing a molecular genetic diagnosis for patients with neonatal/infantile intrahepatic cholestasis (NIIC), and diagnostic rates were varied with various numbers of target genes and patients from a wide range of age.

**What is New**

- The molecular genetic diagnostic rate was 26.6% for patients with NIIC onset below 12 months of age.
- The top 3 diagnoses were of Alagille syndrome, neonatal Dubin-Johnson syndrome, and neonatal intrahepatic cholestasis caused by citrin deficiency, and these disorders accounted for 78.8% of the genetic NIIC in Japan.
- Patients with Crigler-Najjar syndrome presenting with elevated indirect hyperbilirubinemia and mild cholestasis were identified by the inclusion of the *UGT1A1* gene in the NIIC panel.

Genetic liver diseases, such as cystic fibrosis (CF), alpha-1 antitrypsin deficiency, Alagille syndrome (ALGS), and progressive familial intrahepatic cholestasis (PFIC), are important causes of intrahepatic cholestasis, potentially progressing toward fibrosis and cirrhosis (1–4). Most of these disorders present as direct hyperbilirubinemia starting from the neonatal or infantile period, the so called neonatal/infantile intrahepatic cholestasis (NIIC). Rather than physiological, NIIC is a sign of hepatobiliary disorders, and some conditions of sustained cholestasis may lead to a failure to thrive and life-threatening complications such as intracranial hemorrhage from vitamin K deficiency coagulopathy (3, 4). Although the outcomes of NIIC are changing with liver transplantation and emerging drugs, clinicians have difficulty in providing precise diagnosis of the cause, because of similar and broad clinical features. Thus, molecular genetic diagnostics can be crucial to establish a definitive diagnosis. Furthermore, with recent advances in molecular testing, especially with the development of next-generation sequencing (NGS) technology and bioinformatics, molecular genetic diagnoses of NIIC have been expanding (5–11).

Since 2013, we have been using NGS in patients with NIIC. In a previous paper, we reported achieving a definitive molecular diagnosis in 26% of the patients subjected to our targeted 18-gene panel, using the Ion Personal Genome Machine system (Ion PGM system) and bioinformatics pipelines (6). However, approximately 70% of the patients still remained without a definitive etiological diagnosis. Therefore, we decided to reconstruct a new diagnostic targeted 61-gene panel–the advanced NIIC gene panel, to improve the diagnostic rate for cholestatic patients with disease onset below 12 months of age.

The aim of the current study was to ascertain the efficacy of the advanced NIIC gene panel and our NGS system by analyzing 2 cohorts of 315 patients in total with NIIC in a clinical setting; one group was retrospectively subjected to a second genetic analysis and the other was prospectively exposed to molecular testing for the first time.

## METHODS

All suspected patients with genetic liver diseases were recruited from 150 hospitals in Japan between May 2013 and October 2019. Serum direct hyperbilirubinemia is the most common marker of cholestasis and in the current study, we defined cholestasis as a serum direct bilirubin (D.Bil) level > 1.0 mg/dL (13). Patients with NIIC were recruited based on the following inclusion criteria: (1) cholestasis, (2) onset < 12 months of age, and (3) no definitive molecular diagnosis previously. We excluded patients with a diagnosis of extrahepatic cholestasis, such as biliary atresia, and chromosomal abnormalities. We also excluded patients born before January 2010 on the suspicion of less precise clinical and laboratory data. Our study protocol complied with the ethical guidelines of the 1964 Declaration of Helsinki (2013 revision) and was approved by the Ethical Committee of Nagoya City University Graduate School of Medical Sciences.

### Retrospective Reanalysis Group

**FIGURE 1.**
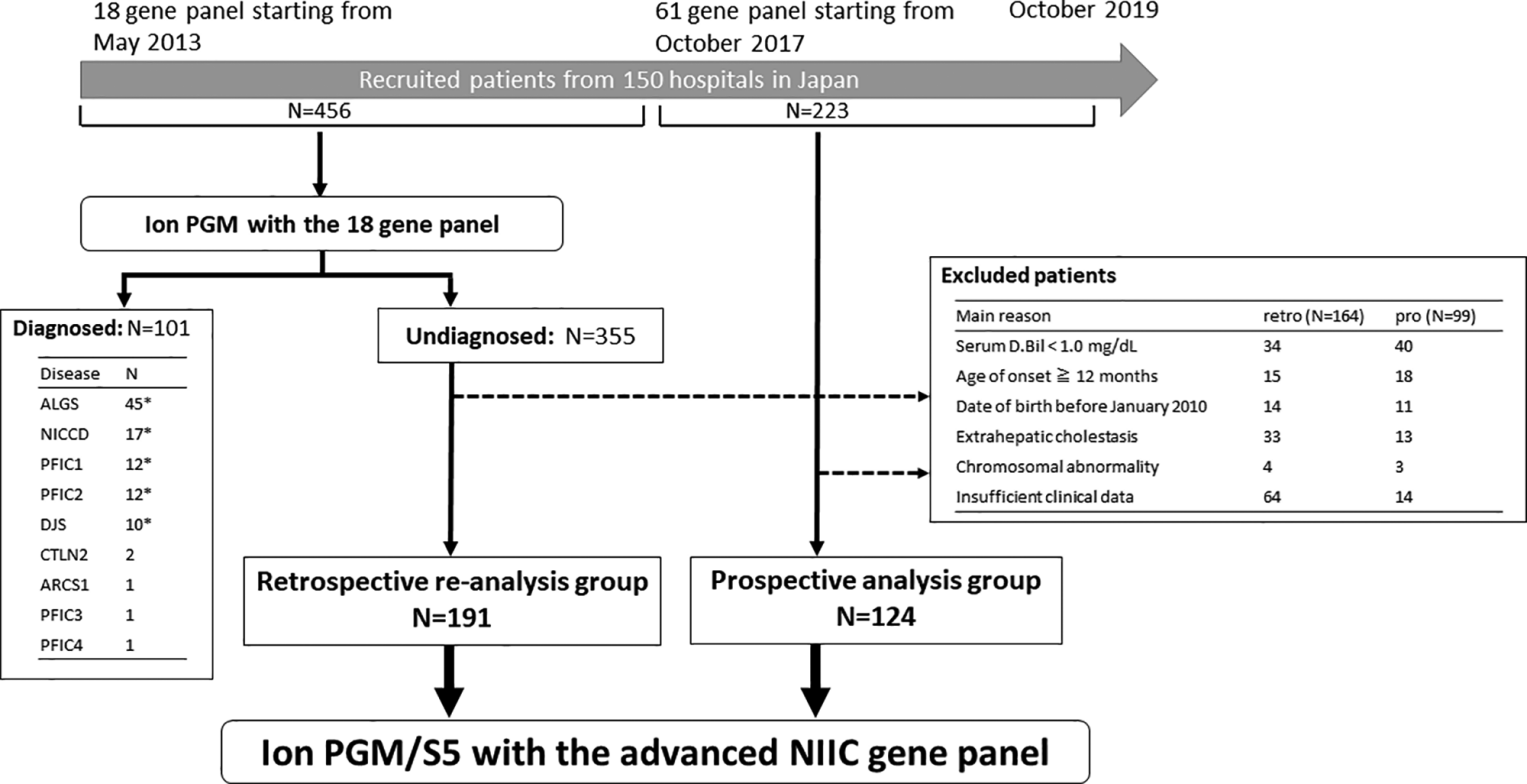
Flow diagram of the current study. ALGS = Alagille syndrome; ARCS = arthrogryposis-renal dysfunction-cholestasis syndrome; CTLN2 = adult-onset type II citrullinemia; D.Bil = direct bilirubin; DJS = Dubin-Johnson syndrome; Ion PGM = Ion Personal Genome Machine system; Ion S5 = Ion GeneStudio S5 system; NICCD = neonatal intrahepatic cholestasis caused by citrin deficiency; NIIC = neonatal/infantile intrahepatic cholestasis; PFIC = Progressive familial intrahepatic cholestasis. *12 of ALGS, 5 of NICCD, 2 of PFIC type 1, 4 of PFIC type 2, and 5 of DJS were reported in our previous study (6).

A total of 456 patients were identified for the retrospective reanalysis group, from May 2013 to September 2017. Of them, 101 were excluded because of a definitive molecular diagnosis with the old 18-gene panel. The diagnoses of the 101 patients mainly consisted of ALGS, neonatal intrahepatic cholestasis caused by citrin deficiency (NICCD), PFIC type 1, PFIC type 2, and Dubin-Johnson syndrome (DJS) at frequencies of 45, 17, 12, 12, and 10, respectively. We then excluded a further 164 patients from the remaining, previously undiagnosed 355 patients due to serum D.Bil level < 1.0 mg/dL, age of onset ≥ 12 months, diagnosis of extrahepatic cholestasis, or insufficient clinical data (Fig. 1). Finally, 191 patients were enrolled in the retrospective reanalysis group. Thus, we sought to uncover a molecular diagnosis by reanalysis using the advanced NIIC gene panel.

### Prospective Analysis Group

We prospectively recruited 223 patients from October 2017 to October 2019, and of them, 124 were determined to be suitable for the prospective analysis group, according to the same inclusion and exclusion criteria as the retrospective reanalysis group. These patients did not undergo any previous molecular genetic analysis and did not have a definitive molecular diagnosis before the current study. Thus, the 124 patients in this cohort were tested with the advanced NIIC gene panel (Fig. 1). In this group, we aimed to compare the rate of successful molecular diagnosis with the advanced panel, to that reported in our previous study.

### The Advanced NIIC Gene Panel, Preparation of the Library and Sequencing

The advanced NIIC gene panel contained 61-genes associated with intrahepatic cholestasis, including *JAG1* and *NOTCH2* associated with ALGS type 1 and 2 respectively; *ATP8B1*, *ABCB11*, *ABCB4*, *TJP2*, and *NR1H4* associated with PFIC type 1 to 5, respectively; and *ABCC2* associated with DJS. Because jaundice is an important symptom of neonatal cholestasis, and as the prevalence of Gilbert syndrome (GS) is 12.5% in Japan (14), we included *UGT1A1*, a candidate gene (the expression of which leads to indirect hyperbilirubinemia) known to cause constitutional jaundice upon expression, and the causative gene of Crigler-Najjar syndrome (CNS) type 1, CNS type 2, and GS. All of the 61-genes included in the advanced NIIC panel are listed in Supplemental Digital Content Table 1 (http://links.lww.com/PG9/A78).

The amplicon library of the advanced NIIC gene panel was designed using the Ion AmpliSeq designer (version 6.0; Thermo Fisher Scientific, Waltham, MA) in July 2017. The number of exons, amplicons, and total targeted bases were 998, 1,387, and 155,387, respectively. This new NIIC panel ensured a theoretical coverage of 99.0% of the target sequences. DNA extraction and library construction were performed as previously described (6). NGS was performed using either the Ion PGM system or the Ion GeneStudio S5 system (Thermo Fisher Scientific).

### Variant Detection Pipeline, Validation Analysis, and Molecular Genetic Diagnosis

Sequence data analysis pipelines were established as described in our previous report (6). Minor allele frequency (MAF) was obtained from the Japanese dataset of the Human Genetic Variation Database (HGVD; https://www.hgvd.genome.med.kyoto-u.ac.jp/) and the Genome Aggregation Database (gnomAD; https://gnomad.broadinstitute.org/). We adopted the Combined Annotation Dependent Depletion (https://cadd.gs.washington.edu/) tool for computational prediction of potential pathogenicity of the variants. Detected variants were classified into 5 categories on the basis of the American College of Medical Genetics and Genomics (ACMG) guidelines: pathogenic, likely pathogenic, uncertain significance, likely benign, and benign (15).

For autosomal recessive variants in the *UGT1A1* gene, encoding UDP-glucuronosyltransferase (UGT1A1), we not only assessed pathogenic variants, but also functional polymorphisms determined to be benign as per ACMG guidelines, which can cause CNS and GS in Japanese patients (16). In the current study, presence of biallelic null variants (0% of UGT1A1 activity) was diagnosed as CNS type 1, biallelic variants leading to severe reduction of UGT1A1 activity as per previous reports (≤10% at homozygous state of variants) or a combination of an allele causing severe reduction with another causing mild reduction (UGT1A1 activity ≤ 50%) as CNS type 2, and biallelic mild reduction as GS (17–19).

### Statistics

The values from the clinical and laboratory findings of the 2 groups were analyzed statistically using the χ^2^ method or the Wilcoxon signed-rank test. Statistical analyses were performed with JMP 11.2 (SAS Institute, Cary, North Carolina), and a *P* value <0.05 was chosen as the cutoff for significance.

## RESULTS

### Patient Characteristics

In the retrospective reanalysis group, median age of onset of cholestasis and age at the time of this study were 1 month [0–2, interquartile range (IQR)] and 46 months (33–64, IQR), respectively. The median values of serum total bilirubin (T.Bil)/D.Bil, aspartate aminotransferase (AST)/alanine aminotransferase (ALT), γ-glutamyltransferase (GGT), and total bile acids (TBA) levels were 7.3/4.0 mg/dL, 121/65 IU/L, 97 IU/L, and 114 µmol/L, respectively. In the prospective analysis group, median age of onset of cholestasis and age at the time of this survey were 0 month (0–1, IQR) and 4 months (3–6, IQR), respectively. The median values of serum T.Bil/D.Bil, AST/ALT, GGT, and TBA were 7.5/4.4 mg/dL, 133/59 IU/L, 121 IU/L, and 135 µmol/L, respectively. The age of onset, age at the time of NGS, and TBA levels were statistically different between both groups. The patient characteristics of both groups are detailed in Table 1.

**TABLE 1.** Patient characteristics of retrospective reanalysis group and prospective analysis group

	Retro (N = 191)		Pro (N = 124)		
	N (%)		N (%)		*P*
Male	113 (59.2)		74 (59.7)		0.9
Age at onset <1 month	77 (39.3)		65 (52.4)		0.02
Liver biopsy	41 (21.5)		31 (25.0)		0.5
GA <37 weeks	55 (29.9)*		38 (31.4)†		0.8
SGA	49 (26.6)*		33 (27.3)†		1.0
Sepsis	3 (1.6)		0 (0)		0.08
	**N**	**Median (IQR)**	**N**	**Median (IQR)**	**P**
GA, week	184	38 (36–39)	121	38 (36–39)	0.3
BW, g	184	2601 (2005–3061)	124	2565 (1816–2949)	0.5
Age at onset, month	191	1 (0–2)	124	0 (0–1)	<.01
Age at NGS, month	189	46 (33–64)	124	4 (3–6)	<.01
T.Bil, mg/dL	189	7.3 (5.2–10.4)	124	7.5 (5.6–11.5)	0.5
D.Bil, mg/dL	191	4.0 (2.4–6.1	124	4.4 (2.8–7.2)	0.09
AST, IU/L	189	121 (58–232)	124	133 (67–234)	0.6
ALT, IU/L	189	65 (30–168)	124	59 (27–136)	0.7
GGT, IU/L	187	97 (61–227)	123	121 (60–229)	0.7
TBA, μmol/L	169	114 (75–176)	113	135 (95–234)	0.01

*184 patients were collected.

†121 patients were collected.

ALT = alanine aminotransferase; AST = aspartate aminotransferase; BW = birth weight; D.Bil = direct bilirubin; GA = gestational age; GGT = γ-glutamyltransferase; IQR = interquartile range; Pro = prospective analysis group; Retro = retrospective reanalysis group; SGA = small for gestational age; T.Bil = total bilirubin; TBA = total bile acids.

### Sequencing Data Summary

The median number of total sequenced bases per patient, median number of mapped reads, and mean read length in the retrospective reanalysis group and the prospective analysis group were 96.6 (86.2–108, IQR) and 95.5 mega bases (82.3–116, IQR), 503 (441–563, IQR), and 538 kilo reads (449–623, IQR), and 196 (188–198, IQR), and 188 (173–194, IQR), respectively. The mean depth of coverage in the target regions in the retrospective reanalysis group and the prospective analysis group was 322-fold (281–336, IQR) and 316-fold (264–380, IQR), respectively, and 95.6% and 94.3% of the target regions had more than 100-fold coverage, respectively.

### Molecular Genetic Diagnosis of NIIC

#### Retrospective Reanalysis Group

In the retrospective reanalysis group, with our sequencing and bioinformatics analyses, 10 (5.2%) patients received a definitive molecular genetic diagnosis; they carried causative variants in *ABCC2*, *MPV17*, *NPC1*, *CFTR*, *NR1H4*, or *CYP27A1* (Table 2). Table 3 shows the pathogenic and likely pathogenic variants detected in the patients. Two patients (patients r-4 and r-5 in Table 3) had compound heterozygous pathogenic or likely pathogenic variants in *ABCC2*. Of those 4 variants, p.(Arg393Trp) and c.1967 + 2T>C in heterozygous state, were previously detected in the old 18-gene panel in patients r-4 and r-5, respectively. The other variants, c.633-275_867 + 498del and p.(Ala44Asp) respectively, were newly recognized with the advanced NIIC gene panel, and we were finally able to diagnose these 2 patients with neonatal DJS (nDJS). The other 5 genes in which variants were detected, *MPV17*, *NR1H4*, *NPC1*, *CYP27A1,* and *CFTR*, were not included in the old 18-gene panel. Thus, we diagnosed 10 patients in total in this cohort by reanalysis using the advanced NIIC gene panel as follows: 3 patients were diagnosed with mitochondrial DNA depletion syndrome, 2 with nDJS, 2 with PFIC type 5, one with Niemann-Pick disease type C (NPC), one with cerebrotendinous xanthomatosis and one with CF. Supplemental Digital Content Table 2 (http://links.lww.com/PG9/A78) presents additional clinical information about the aforementioned 10 patients with molecular diagnoses.

**TABLE 2.** Molecular genetic diagnosis of neonatal/infantile intrahepatic cholestasis

Genetic diagnosis	Affected gene	Retro (N = 191), N (%)	Pro (N = 124), N (%)
ALGS	JAG1*/NOTCH2*	0	10 (8.1)
DJS	*ABCC2* *	2 (1.0)	9 (7.3)
NICCD	*SLC25A13* *	0	7 (5.6)
PFIC2/BRIC2	*ABCB11* *	0	2 (1.6)
Mitochondrial DNA depletion syndrome	*MPV17*	3 (1.6)	0
	*POLG*	0	1 (0.8)
NPC	*NPC1*	1 (0.5)	1 (0.8)
Cystic fibrosis	*CFTR*	1 (0.5)	1 (0.8)
PFIC1/BRIC1	*ATP8B1* *	0	1 (0.8)
PFIC3	*ABCB4* *	0	1 (0.8)
PFIC5	*NR1H4*	2 (1.0)	0
Cerebrotendinous xanthomatosis	*CYP27A1*	1 (0.5)	0
Total		10 (5.2)	33 (26.6)

*Both of advanced NIIC panel and old 18-gene panel contained genes with the symbol.

ALGS = Alagille syndrome; BRIC = benign recurrent intrahepatic cholestasis; DJS = Dubin-Johnson syndrome; NICCD = neonatal intrahepatic cholestasis caused by citrin deficiency; NPC = Niemann-Pick disease type C.; PFIC = progressive familial intrahepatic cholestasis; Pro = prospective analysis group; Retro = retrospective reanalysis group.

**TABLE 3.** Molecular genetic diagnosis of neonatal/infantile intrahepatic cholestasis and variant characteristics

Patients	Genetic diagnosis	Affected gene	Nucleotide change	Predicted amino acid change	Zygosity	ACMG	
						Classification	Evidence
*Retrospective reanalysis group*							
r-1	Mitochondrial DNA depletion syndrome	*MPV17*	c.148C>T	p.(Arg50Trp)	Hetero	LP	PS3, PM2, PM3, PP3
		*MPV17*	c.149G>A	p.(Arg50Gln)	Hetero	LP	PS3, PM2, PM3, PP3
r-2	Mitochondrial DNA depletion syndrome	*MPV17*	c.451dup	p.(Leu151Profs*39)	Homo	P	PVS1, PM2, PM3
r-3	Mitochondrial DNA depletion syndrome	*MPV17*	c.308_310del	p.(Cys103del)†	Hetero	LP	PM2, PM3, PM4, PP3
		*MPV17*	c.451dup	p.(Leu151Profs*39)	Hetero	P	PVS1, PM2, PM3
r-4	DJS	*ABCC2*	c.1177C>T	p.(Arg393Trp)‡	Hetero	LP	PM2, PM3, PP1, PP3
		*ABCC2*	c.633-275 _867 + 498del	Exon 7 deletion	Hetero	P	PVS1, PM3, PP1
r-5	DJS	*ABCC2*	c.131C>A	p.(Ala44Asp)†	Hetero	LP	PM2, PM3, PP1, PP3
		*ABCC2*	c.1967 + 2T>C‡	Splice-site disruption	Hetero	P	PVS1, PM2, PM3, PP1
r-6	PFIC5	*NR1H4*	c.526C>T	p.(Arg176*)	Hetero	P	PVS1, PM2, PP1
		*NR1H4*	c.1066 + 5G>C†	Splice-site disruption	Hetero	LP	PM2, PM3, PP1, PP3
r-7	PFIC5	*NR1H4*	c.862-2A>G†	Splice-site disruption	Homo	P	PVS1, PM2, PM3
r-8	NPC	*NPC1*	c.864del	p.(Phe288Leufs*22)†	Hetero	P	PVS1, PM2, PM3
		*NPC1*	c.3618del	p.(Lys1206Asnfs*36)	Hetero	P	PVS1, PM2, PM3
r-9	Cerebrotendinous xanthomatosis	*CYP27A1*	c.245A>T	p.(His82Leu)†	Hetero	LP	PM2, PM3, PP1, PP3
		*CYP27A1*	c.435G>T	p.(=) (splice-site disruption)	Hetero	LP	PS3, PM2, PP1
r-10	Cystic fibrosis	*CFTR*	c.3909C>G	p.(Asn1303Lys)	Hetero	LP	PS3, PM2, PM3, PP3
		*CFTR*	c.4054C>T	p.(Gln1352*)†	Hetero	P	PVS1, PM2, PM3
***Prospective analysis group***							
p-1	ALGS	*JAG1*	c.2698C>T	p.(Arg900*)	Hetero	P	PVS1, PM2, PP4
p-2	ALGS	*JAG1*	c.760C>T	p.(Gln254*)†	Hetero	P	PVS1, PM2, PP1, PP4
p-3	ALGS	*JAG1*	c.1372dup	p.(Cys458Leufs*4)†	Hetero	P	PVS1, PM2, PP4
p-4	ALGS	*JAG1*	c.1313G>A	p.(Cys438Tyr)†	Hetero	LP	PM2, PM5, PP1, PP3, PP4
p-5	ALGS	*JAG1*	c.1977G>A	p.(Trp659*)	Hetero	P	PVS1, PM2, PP4
p-6	ALGS	*JAG1*	g.(?_10620123)_(10654301_?)del	Whole exon deletion	Hetero	P	PVS1, PM6, PP4
p-7	ALGS	*JAG1*	c.1570-21C>A†¶	p.(Asp525Phefs*12)	Hetero	P	PVS1, PS3, PM2, PP1, PP4
p-8	ALGS	*JAG1*	c.1457dup	p.(Asp487Argfs*4)†	Hetero	P	PVS1, PM2, PP1
p-9	ALGS	*JAG1*	c.1721-1G>C	Splice-site disruption	Hetero	P	PVS1, PM2, PP1
p-10	ALGS	*NOTCH2*	c.6027 + 1del†	Splice-site disruption	Hetero	LP	PVS1, PM2
p-11	DJS	*ABCC2*	c.2302C>T	p.(Arg768Trp)	Hetero	P	PS3, PM2, PM3, PP1, PP3
		*ABCC2*	c.2439 + 2T>C	Splice-site disruption	Hetero	P	PVS1, PM2, PM3, PP1
p-12	DJS	*ABCC2*	c.1967 + 2T>C	Splice-site disruption	Hetero	P	PVS1, PM2, PM3, PP1
		*ABCC2*	c.3614 + 1G>A†	Splice-site disruption	Hetero	P	PVS1, PM2, PM3, PP1
p-13	DJS	*ABCC2*	c.2302C>T	p.(Arg768Trp)	Hetero	P	PS3, PM2, PM3, PP1, PP3
		*ABCC2*	c.2951T>G	p.(Leu984Arg)†	Hetero	LP	PM2, PM3, PP1, PP3
p-14	DJS	*ABCC2*	c.1967 + 2T>C	Splice-site disruption	Hetero	P	PVS1, PM2, PM3, PP1
		*ABCC2*	c.2882A>G	p.(Lys961Arg)	Hetero	LP	PM2, PM3, PP1, PP3
p-15	DJS	*ABCC2*	c.2302C>T	p.(Arg768Trp)	Homo	P	PS3, PM2, PM3, PP1, PP3
p-16	DJS	*ABCC2*	c.1967 + 2T>C	Splice-site disruption	Hetero	P	PVS1, PM2, PM3, PP1
		*ABCC2*	c.3928C>T	p.(Arg1310*)	Hetero	P	PVS1, PM2, PM3
p-17	DJS	*ABCC2*	c.2125T>C	p.(Trp709Arg)	Hetero	P	PS3, PM2, PM3, PP1, PP3
		*ABCC2*	c.4081G>A	p.(Asp1361Asn)†	Hetero	LP	PM2, PM3, PP1 PP3
p-18	DJS	*ABCC2*	c.2882A>G	p.(Lys961Arg)	Hetero	LP	PM2, PM3, PP1, PP3
		*ABCC2*	c.3928C>T	p.(Arg1310*)	Hetero	P	PVS1, PM2, PM3
p-19	DJS, GS§	*ABCC2*	c.2125T>C	p.(Trp709Arg)	Hetero	P	PS3, PM2, PM3, PP1, PP3
		*ABCC2*	c.2439 + 2T>C	Splice-site disruption	Hetero	P	PVS1, PM2, PM3, PP1
		*UGT1A1*	c.-41_-40dup	Promoter disruption	Homo	B	BA1
p-20	NICCD	*SLC25A13*	c.852_855del	p.(Met285Profs*2)	Hetero	P	PVS1, PM3, PP1
		*SLC25A13*	c.1311 + 1G>A	Splice-site disruption	Hetero	P	PVS1, PM3, PP1
p-21	NICCD	*SLC25A13*	c.1180 + 1G>A	Splice-site disruption	Hetero	P	PVS1, PM3, PP1
		*SLC25A13*	c.1595G>A	p.(Gly532Asp)	Hetero	LP	PS3, PM3, PP1, PP3
p-22	NICCD	*SLC25A13*	c.852_855del	p.(Met285Profs*2)	Homo	P	PVS1, PM3, PP1
p-23	NICCD	*SLC25A13*	c.852_855del	p.(Met285Profs*2)	Hetero	P	PVS1, PM3, PP1
		*SLC25A13*	c.1180 + 1G>A	splice-site disruption	Hetero	P	PVS1, PM3, PP1
p-24	NICCD	*SLC25A13*	c.852_855del	p.(Met285Profs*2)	Hetero	P	PVS1, PM3, PP1
		*SLC25A13*	c.1311 + 1G>A	splice-site disruption	Hetero	P	PVS1, PM3, PP1
p-25	NICCD, GS§	*SLC25A13*	c.852_855del	p.(Met285Profs*2)	Homo	P	PVS1, PM3, PP1
		*UGT1A1*	c.211G>A	p.(Gly71Arg)	Homo	B	BA1
p-26	NICCD, GS§	*SLC25A13*	c.1180 + 1G>A	Splice-site disruption	Hetero	P	PVS1, PM3, PP1
		*SLC25A13*	c.1595G>A	p.(Gly532Asp)	Hetero	LP	PS3, PM3, PP1, PP3
		*UGT1A1*	c.211G>A	p.(Gly71Arg)	Homo	B	BA1
p-27	PFIC2	*ABCB11*	c.1907A>G	p.(Glu636Gly)	Hetero	LP	PM2, PM3, PP1, PP3
		*ABCB11*	c.2782C>T	p.(Arg928*)	Hetero	P	PVS1, PM2, PM3
p-28	PFIC2/BRIC2	*ABCB11*	c.1723C>T	p.(Arg575*)	Hetero	P	PVS1, PM2, PP1
		*ABCB11*	c.3011G>T	p.(Gly1004Val)†	Hetero	LP	PM2, PM3, PM5, PP1, PP3
p-29	PFIC1/BRIC1	*ATP8B1*	c.627 + 1G>A†	Splice-site disruption	Hetero	P	PVS1, PM2, PM3
		*ATP8B1*	c.3596_3601delinsT	p.(Arg1199Leufs*35)†	Hetero	P	PVS1, PM2, PM3
p-30	PFIC3	*ABCB4*	c.461T>C	p.(Phe154Ser)	Hetero	LP	PM2, PM3, PP1, PP3
		*ABCB4*	c.2177C>T	p.(Pro726Leu)	Hetero	P	PS3, PM2, PM3, PP1, PP3
p-31	Mitochondrial DNA depletion syndrome	*POLG*	c.2890C>T	p.(Arg964Cys)	Homo	LP	PS3, PM3, PP1, PP3
p-32	NPC	*NPC1*	c.3277A>G	p.(Thr1093Ala)†	Hetero	LP	PM2, PM3, PP1, PP3
		*NPC1*	c.3618del	p.(Lys1206Asnfs*36)	Hetero	P	PVS1, PM2, PP1
p-33	Cystic fibrosis	*CFTR*	c.2908 + 1085_3367 + 260del	Exon 18-20 deletion	Homo	P	PVS1, PM3, PP1

†Novel pathogenic variant.

‡Those 2 variants were reported in our previous study (6).

§Patients molecularly diagnosed as coexistence of a genetic cholestasis and Gilbert syndrome.

¶We performed a splicing analysis by cDNA sequence.

NCBI reference sequences: *ABCB11*, *NM_003742.4*; *ABCB4*, *NM_000443.4*; *ABCC2*, *NM_000392.5*; *ATP8B1*, *NM_005603.6*; *CFTR*, *NM_000492.4*; *CYP27A1*, *NM_000784.4*; *JAG1*, *NM_000214.3*; *MPV17*, *NM_002437.5*; *NOTCH2*, *NM_024408.4*; *NPC1*, *NM_000271.5*; *NR1H4*, *NM_005123.4*; *POLG*, *NM_002693.3*; *SLC25A13*, *NM_001160210.1*; *UGT1A1*, *NM_000463.3*.

#### Prospective Analysis Group

In the prospective analysis group, we were able to determine a molecular diagnosis for 33 (26.6%) of the 124 patients; our analyses indicated that they harbored variants in *JAG1*, *NOTCH2*, *ABCC2*, *SLC25A13*, *ABCB11*, *POLG*, *NPC1*, *CFTR*, *ATP8B1*, or *ABCB4* (Table 2). The median age at the time of the NGS analysis was 4 months in the 33 patients that received a definitive molecular diagnosis. Ten patients received a genetic diagnosis of ALGS, which was the most frequent diagnosis in the prospective analysis group. Further, 9 patients were diagnosed with nDJS and 7 with NICCD. ALGS, nDJS, and NICCD together accounted for 78.8% of all genetic diagnoses of NIIC made in Japan. Detailed information on the detected variants is listed in Table 3 and additional clinical information is presented in Supplemental Digital Content Table 2 (http://links.lww.com/PG9/A78). Of the 10 patients molecularly diagnosed with ALGS, 7 fully satisfied the clinical criteria for ALGS, 2 were clinically suspected to have ALGS, and one was suspected to have nDJS before the analysis. Three of the detected genes, *POLG*, *NPC1*, and *CFTR*, were not included in the old 18-gene panel and patients harboring variants in them were molecular diagnosed with mitochondrial DNA depletion syndrome, NPC and CF, respectively. Twenty of the causative variants identified in both groups were not registered in the human gene mutation database or any other public database of variants; therefore, these variants were considered to be novel pathogenic variants (Table 3).

### Pathogenic or Likely Pathogenic Variants on a Single Allele

In 20 patients of the retrospective reanalysis group and 17 of the prospective analysis group, only a single heterozygous pathogenic or likely pathogenic variant, in one of the genes normally associated with an autosomal recessive disease was identified. In both groups, we identified 16 genes, of which *ABCB11*, *SLC25A13*, and *ABCC2* were most common; their frequencies were 12, 5, and 5, respectively (Supplemental Digital Content Table 3, http://links.lww.com/PG9/A78). Detailed information on the detected variants and phenotypes is presented in Supplemental Digital Content Table 4 (http://links.lww.com/PG9/A78).

### CNS and GS Among the Patients in the Study

Concerning the *UGT1A1* gene, we detected 3 patients in the retrospective reanalysis group (r-12, r-13, and r-14 in Supplemental Digital Content Table 5, http://links.lww.com/PG9/A78) with genotypes associated with CNS type 2. These 3 patients showed severe indirect hyperbilirubinemia and mild cholestasis in the first few months of life; serum T.Bil/D.Bil levels were 24.0/1.3, 19.7/1.4, and 24.3/2.4 mg/dL in each patient. In patient r-12, we detected the double missense variants p.[(Gly71Arg; Tyr486Asp), which were located on the same allele, and the missense variant p.(Arg209Trp). Further, we detected p.[(Gly71Arg; Tyr486Asp)] and p.(Gly71Arg) in patient r-13, and p.(Cys280*) and p.(Gly71Arg) in patient r-14. We did not identify any genotype associated with CNS type 1.

We identified genotypes associated with GS in 32 patients, of which, 21 belonged to the retrospective reanalysis group and 11 were in the prospective analysis group. The genotypes of these patients are listed in Supplemental Digital Content Table 6 (http://links.lww.com/PG9/A78): 21 patients were homozygous for p.(Gly71Arg); 7 patients were 2 heterozygous variants p.(Gly71Arg) and TA-insertion mutation in the TATA box (c.-41_-40dup); 2 patients were 2 heterozygous variants p.(Pro229Gln) and c.-41_-40dup; and 2 patients were homozygous for c.-41_-40dup. It is noteworthy that 3 patients were molecularly diagnosed with a coexistence of NICCD with GS (p-19 and p-25) and nDJS with GS (p-26), and they had genetic cholestasis and indirect hyperbilirubinemia.

## DISCUSSION

In the current study, we analyzed 2 cohorts of patients with NIIC–a retrospective reanalysis group, and a prospective analysis group to evaluate the efficacy of the reconstructed, advanced NIIC gene panel. In the retrospective reanalysis group, we newly determined the etiology of NIIC in 10 of 191 patients (5.2%). Eight of the 10 patients carried causative variants in genes that were not included in our old gene panel: *MPV17*, *NPC1*, *CFTR*, *NR1H4*, and *CYP27A1*. Additionally, in 2 of the 10 patients, we were able to find previously undetected variants of the *ABCC2* gene; they were then newly diagnosed with nDJS. The 2 variants were a deletion extending from intron 6 to intron 7 and a missense variant in exon 2. Our new sequencing system improved both sequenced read length and depth of coverage, on the location of exons 2 and 7 in *ABCC2*. Thus, we concluded that our advanced NIIC panel worked more efficiently than our old 18-gene panel, owing to the number of target genes, as well as the wider range and precise sequencing of the target sequences.

In the prospective analysis group, a definitive molecular genetic etiology was identified for 33 of the 124 patients (26.6%) in 10 genes; 10 diagnoses were of ALGS, 9 of nDJS, and 7 of NICCD (8.1%, 7.3%, and 5.6%, respectively). We observed that the molecular genetic diagnostic rate in the prospective analysis group (26.6%) was similar to that in our previous study (25.7%), as well as to other reports (6, 8, 10, 12). This might be attributable to 2 factors. First, newly detected genes in the advanced NIIC panel, such as *POLG*, *NPC1*, or *CFTR*, are extremely rarely affected in Japan (20–22). In our study, 3 genetic diseases, ALGS, nDJS, and NICCD, accounted for 78.8% (22/36) of the molecular diagnoses, similar to our previous report (6). Therefore, even though we identified patients in whom *POLG*, *NPC1*, or *CFTR* are affected, these conditions would not contribute to improved diagnostic yield in the NIIC setting in Japan. Second, the diagnostic rate might largely depend on the selection of patients. Because the etiology of NIIC not only depends on genetic defects and their nature, but also on nongenetic complications such as sepsis, viral infections, or perinatal abnormalities like prematurity and asphyxia, the number of genes in a diagnostic panel may not be correlated with the rate of success in establishing a molecular etiological diagnosis of neonatal intrahepatic cholestasis in Japan (10). Nevertheless, the diagnosis yield might vary between cohorts because of potential selection bias due to the time of collection in the current and previous studies.

We found 37 patients (20 in the retrospective reanalysis group and 17 in the prospective analysis group) with a single heterozygous pathogenic or likely pathogenic variant in one of the genes associated with an autosomal recessive disease, such as *ABCB11.* We identified a pathogenic or a likely pathogenic variant on a single allele of *ABCB11* in 12 patients, but could only molecularly confirm PFIC type 2 in 2 patients. This indicates that either another mutation remained unidentified in the gene, or a single heterozygous alteration might predispose to cholestasis (23). Whole genome sequencing (WGS) could detect variants of the promoter region and other structural variants, while whole transcriptome analysis can identify an abnormal mRNA and consequently uncover an aberrant splice site (24, 25). These have the potential to help in the diagnosis of patients with an unidentified etiology. Among patients with autism spectrum disorder, a few cases with structural variants in the causative gene were detected by WGS (26). Whole exome sequencing (WES) might not contribute to a visibly improved molecular diagnostic rate, considering that ALGS, nDJS, and NICCD constitute approximately 80% of all the genetic causes of NIIC in Japan. Nevertheless, WES could expose an extremely rare NIIC disorder in a clinical setting or discover a new candidate gene of cholestasis in the research field (9).

We identified 3 patients with genotypes associated with CNS in the retrospective reanalysis group. These patients had 2 or more causative variants of CNS or GS and showed severe indirect hyperbilirubinemia and mild cholestasis in the first few months of life. Our data suggest that clinicians need to consider including patients with CNS in the cohort of NIIC, especially during the neonatal period when we define cholestasis as serum D.Bil level > 1.0 mg/dL (13). Regarding detected GS genotypes in the *UGT1A1* gene, we molecularly diagnosed 21 of 191 (11.0%) and 11 of 124 (8.9%) patients with GS in the retrospective reanalysis group and the prospective analysis group, respectively. Our detection rates of the GS genotype were almost the same as those previously reported in the Japanese population (14). We presumed that those patients had other causes of intrahepatic cholestasis, because GS commonly presents with mild indirect hyperbilirubinemia and the patients, we diagnosed with GS had direct hyperbilirubinemia; median levels of serum T.Bil/D.Bil were 8.4/3.4 mg/dL (16).

Finally, we discuss the genes that should be launched for the genetic analysis of patients with NIIC. In our previous study, we created an 18-gene panel that included genes responsible for genetic diseases that are relatively common in East Asia (6). However, after the start of the study, we received requests for genetic analysis from patients of non-East Asian ethnicity, such as Latin American, Southeast Asian, and European patients who lived in Japan. Therefore, in this study, we reconstructed the 61-gene panel by increasing the number of causative genes without regard to race and our 61-gene panel was similar to other available panels (5, 7, 8, 11). In fact, in this study, we identified patients with CF, which is extremely rare in Japan. In clinical practice, a universally available panel would be more useful than an ethnic panel for the early detection and timely treatment of rare mitochondrial diseases and Niemann-Pick type C, even in an East Asian region like Japan.

In conclusion, the advanced NIIC gene panel successfully uncovered molecular genetic diagnoses in not only the reanalysis group, but also prospectively included patients in the clinical setting; extremely rare causative genes such as *NR1H4* or *CYP27A1*, as well as commonly implicated causes of NIIC like *JAG1*, *ABCC2*, and *SLC25A13* were identified and our detection rate was 26.6% in the prospective cohort. Clinicians should not forget that patients with CNS type 2 exist in the cohort with direct hyperbilirubinemia. Further studies using WGS and RNA sequence analysis are needed to confirm a definitive molecular diagnosis or to find new candidate genes of NIIC, especially for patients in whom we could only identify one pathogenic variant on a single allele.

## ACKNOWLEDGMENTS

We wish to thank the patients and their families for their participation in the current study, as well as the clinicians for providing patient samples and information (Supplemental Digital Content Table 7, http://links.lww.com/PG9/A78). We also thank the Core Laboratory, Nagoya City University Graduate School of Medical Sciences, and the University of Tsukuba Hospital.

## Supplementary Material


