# Supplementary material for: Real-life Progression of the Use of a Genetic Panel in to Diagnose Neonatal Cholestasis
Source: JPGN Rep. 2022 Mar 31;3(2):e196. doi: 10.1097/PG9.0000000000000196 (PMC10158323; doi:10.1097/PG9.0000000000000196)
Supplement: Supplementary file 1 [file pg9-3-e196-s001.pdf]

Table, Supplemental Digital Content 1. The gene list of the advanced NIIC gene panel

Disorder	Gene name
PFIC	*ATP8B1**, *ABCB11**, *ABCB4**, *TJP2**, *NR1H4*
ARCS	*VPS33B**, *VIPAS39*
Dubin-Johnson syndrome	*ABCC2**
Rotor syndrome	*SLCO1B1*, *SLCO1B3*
Cystic fibrosis	*CFTR*
Neonatal sclerosing cholangitis	*CLDN1*
Zellweger syndrome	*PEX1*, *PEX2*, *PEX3*, *PEX5*, *PEX6*, *PEX10*, *PEX12*, *PEX13*, *PEX14*, *PEX16*, *PEX19*, *PEX26*
NICCD	*SLC25A13**
Bile acid metabolic defects	*HSD3B7**, *AKR1D1**, *CYP7B1**, *SLC27A5*, *CYP7A1*
Familial hypercholanemia	*BAAT**
Crigler-Najjar syndrome/Gilbert syndrome	*UGT1A1*
Niemann-Pick disease	*NPC1*, *NPC2*
Wilson disease	*ATP7B*
Alpha-1-antitrypsin deficiency	*SERPINA1*
Alpha-methylacyl-CoA racemase deficiency	*AMACR*
Cerebrotendinous xanthomatosis	*CYP27A1*
Galactosemia	*GALT*
Lysosomal acid lipase deficiency	*LIPA*
Smith-Lemli-Opitz syndrome	*DHCR7*
Tyrosinemia, type I	*FAH*
Alagille syndrome	*JAG1**, *NOTCH2**
Joubert syndrome/Meckel syndrome	*CC2D2A*, *MKS1*
Nephronophthisis	*NPHP1*, *INVS*, *NPHP3*, *NPHP4*
Polycystic kidney disease	*PKHD1*, *PKD2*
Polycystic liver disease	*SEC63*, *PRKCSH*
Mitochondrial disorder	*DGUOK*, *MPV17*, *BCS1L*, *POLG*, *TRMU*
Others	*ATP11C*, *HNF1B*

NIIC, neonatal/infantile intrahepatic cholestasis; PFIC, progressive familial intrahepatic cholestasis; ARCS, arthrogryposis-renal dysfunction-cholestasis syndrome; NICCD, neonatal intrahepatic cholestasis caused by citrin deficiency.

* Thirteen symbolled genes were contained in our old gene panel (5).

Table, Supplemental Digital Content 2. Phenotypes of the patients with genetic diagnoses

Patients	Clinical Diagnosis	Affected gene	Genetic Diagnosis	Age at onset (month)	GA < 37 weeks	SGA	Liver findings	Outcome
Retrospective re-analysis group								
r-1	unknown	MPV17	Mitochondrial DNA depletion syndrome	8	-	-	N/A	N/A
r-2	unknown	MPV17	Mitochondrial DNA depletion syndrome	1	-	-	giant cell transformation	N/A
r-3	unknown	MPV17	Mitochondrial DNA depletion syndrome	2	-	-	N/A	N/A
r-4	PFIC suspected	ABCC2	DJS	1	-	-	giant cell transformation	transient cholestasis
r-5	unknown	ABCC2	DJS	0	-	-	N/A	transient cholestasis
r-6	PFIC suspected	NR1H4	PFIC5	2	-	-	decreased expression of BSEP	death
r-7	PFIC suspected	NR1H4	PFIC5	3	-	-	decreased expression of BSEP	LT
r-8	neonatal hemochromatosis	NPC1	NPC	0	-	+	ion deposition	LT
r-9	unknown	CYP27A1	Cerebrotendinous xanthomatosis	0	+	-	intrahepatic biliary hypoplasia	N/A
r-10	unknown	CFTR	CF	0	-	-	mild fibrosis	N/A
Prospective analysis group								
p-1	ALGS	JAG1	ALGS	1	-	-	N/A	N/A
p-2	ALGS	JAG1	ALGS	0	-	-	N/A	N/A
p-3	ALGS	JAG1	ALGS	4	-	-	intrahepatic biliary hypoplasia	LT
p-4	ALGS	JAG1	ALGS	0	+	+	N/A	N/A
p-5	ALGS	JAG1	ALGS	0	-	-	N/A	N/A
p-6	ALGS	JAG1	ALGS	1	-	+	intrahepatic biliary hypoplasia	N/A

p-7	ALGS	JAG1	ALGS	0	+	-	N/A	N/A
p-8	DJS	JAG1	ALGS	0	-	+	N/A	N/A
p-9	ALGS suspected	JAG1	ALGS	1	-	+	N/A	N/A
p-10	ALGS suspected	NOTCH2	ALGS	0	-	+	intrahepatic biliary hypoplasia	N/A
p-11	DJS suspected	ABCC2	DJS	2	-	-	decreased expression of MRP2	N/A
p-12	unknown	ABCC2	DJS	0	-	-	N/A	N/A
p-13	DJS suspected	ABCC2	DJS	0	-	+	N/A	N/A
p-14	unknown	ABCC2	DJS	2	-	+	N/A	N/A
p-15	unknown	ABCC2	DJS	1	+	+	intrahepatic biliary hypoplasia	N/A
p-16	unknown	ABCC2	DJS	0	+	-	N/A	N/A
p-17	unknown	ABCC2	DJS	1	+	-	N/A	N/A
p-18	DJS suspected	ABCC2	DJS	0	-	+	N/A	N/A
p-19	unknown	ABCC2, UGT1A1	DJS, GS	2	-	-	N/A	N/A
p-20	NICCD suspected	SLC25A13	NICCD	0	-	-	N/A	N/A
p-21	NICCD suspected	SLC25A13	NICCD	1	-	+	N/A	N/A
p-22	unknown	SLC25A13	NICCD	0	-	-	N/A	N/A
p-23	NICCD suspected	SLC25A13	NICCD	1	-	-	N/A	N/A
p-24	NICCD suspected	SLC25A13	NICCD	1	-	-	N/A	N/A
p-25	NICCD suspected	SLC25A13, UGT1A1	NICCD, GS	4	-	-	N/A	N/A
p-26	NICCD suspected	SLC25A13, UGT1A1	NICCD, GS	0	-	-	N/A	transient cholestasis
p-27	PFIC suspected	ABCB11	PFIC2	3	-	-	N/A	N/A
p-28	unknown	ABCB11	PFIC2/BRIC2	3	-	-	giant cell transformation	N/A
p-29	PFIC suspected	ATP8B1	PFIC1/BRIC1	5	-	-	N/A	N/A
p-30	PFIC suspected	ABCB4	PFIC3	8	-	-	N/A	N/A
p-31	Mitochondrial disease suspected	POLG	Mitochondrial DNA depletion syndrome	1	-	-	massive necrosis	LT
p-32	unknown	NPC1	NPC	0	-	-	mild fibrosis	N/A

p-33	CF suspected	CFTR	CF	0	-	-	N/A	transient cholestasis

ALGS, Alagille syndrome; BSEP, bile salt export pump; CF, Cystic fibrosis; DJS, Dubin-Johnson syndrome; GA, gestational age; GS, Gilbert syndrome; LT, liver transplantation; MRP2, multi-drug resistance protein 2; N/A, not applicable; NICCD, neonatal intrahepatic cholestasis caused by citrin deficiency; NPC, Niemann-Pick disease type C; PFIC, progressive familial intrahepatic cholestasis; SGA, small for gestational age.

Table, Supplemental Digital Content 3. Pathogenic or likely pathogenic variants on a single allele

Affected gene	Retro	Pro	total
ABCB11	9[a]	3[b]	12
SLC25A13	1	4	5
ABCC2	2[c]	3	5
CYP27A1	2	1	3
NPHP1	2	1	3
CFTR	1	1[b]	2
ATP7B	1[c]	0	1
ABCB4	1	0	1
ATP8B1	0	1	1
BCS1L	1[a]	0	1
CYP7B1	0	1	1
PEX12	1	0	1
SLCO1B1	0	1	1
SLCO1B3	1	0	1
UGT1A1	0	1	1
VPS33B	0	1	1

a, Existence of 2 pathogenic variants on *ABCB11* and *BCS1L* in a patient; b, *ABCB11* and *CFTR*; c, *ABCC2* and *ATP7B* as same.

Retro, Retrospective re-analysis group; Pro, Prospective analysis group.

Table, Supplemental Digital Content 4. Genotypes and Phenotypes of the patients with Pathogenic or likely pathogenic variants on a single allele

patient	clinical diagnosis	Affected gene	Nucleotide change	Predicted amino acid change	ACMG class (Evidence)	age at onset (month)	GA < 37 weeks	SGA	liver biopsy	outcome
Retrospective re-analysis group										
r-14	unknown	ABCB11	c.2482_2489del	p.(Thr828Alafs*3)	LP (PVS1, PM2)	9	-	-	N/A	transient cholestasis
r-15	PFIC suspected	ABCB11	g.(?_169800924)_(169801660_?)del	exon 20-21 deletion	LP (PVS1, PM2)	1	-	-	giant cell transformation	N/A
r-16	PFIC suspected	ABCB11	c.386G>A	p.(Cys129Tyr)	LP (PS3, PM2, PP3)	2	-	-	decreased expression of BSEP	LT
r-17	unknown	ABCB11	c.2343+1dup	splice-site disruption	LP (PVS1, PM2)	0	-	-	N/A	N/A
r-18	unknown	ABCB11	c.989G>A	p.(Trp330*)	LP (PVS1, PM2)	2	-	-	N/A	transient cholestasis
r-19	unknown	ABCB11	c.1621A>G	p.(Ile541Val)	LP (PS3, PM2, PP3)	0	-	-	intrahepatic biliary hypoplasia	N/A
r-20	unknown	ABCB11	c.759del	p.(Ile254Leufs*8)	LP (PVS1, PM2)	1	-	-	N/A	N/A
r-21	unknown	ABCB11	c.3692G>A	p.(Arg1231Gln)	LP (PS3, PM2, PP3)	2	-	-	N/A	transient cholestasis
		BCS1L	c.399del	p.(Glu133Aspfs*25)	LP (PVS1, PM2)					
r-22	PFIC suspected	ABCB11	c.1A>G	p.(Met1Val)	LP (PVS1, PM2)	6	-	-	N/A	transient cholestasis
r-23	unknown	SLC25A13	c.1180+1G>A	splice-site disruption	P (PVS1, PM3, PP1)	4	+	-	giant cell transformation	transient cholestasis
r-24	unknown	ABCC2	c.2439+2T>C	splice-site disruption	P (PVS1, PM2, PM3, PP1)	0	-	-	bridging fibrosis	N/A
r-25	Mitochondrial disease suspected	ABCC2	c.2302C>T	p.Arg768Trp	P (PS3, PM2, PM3, PP1, PP3)	9	-	-	N/A	LT
		ATP7B	c.2513del	p.(Lys838Serfs*35)	LP (PVS1, PM2)					
r-26	unknown	CYP27A1	c.1421G>A	p.(Arg474Gln)	LP (PS3, PM2, PP3)	0	+	+	N/A	N/A
r-27	unknown	CYP27A1	c.1214G>A	p.(Arg405Gln)	LP (PS3, PM2, PP3)	0	+	-	N/A	death
r-28	unknown	NPHP1	g.(?_110881291)_(110962603_?)del	whole exon deletion	LP (PVS1, PM2)	2	-	-	N/A	N/A
r-29	unknown	NPHP1	g.(?_110881291)_(110959111_?)del	whole exon deletion	LP (PVS1, PM2)	1	-	+	N/A	N/A
r-30	unknown	CFTR	c.4045G>A	p.(Gly1349Ser)	LP (PS3, PM2, PP3)	0	+	+	giant cell transformation	N/A
r-31	unknown	ABCB4	c.475C>T	p.(Arg159*)	LP (PVS1, PM2)	4	-	+	N/A	transient cholestasis
r-32	unknown	PEX12	c.268_269del	p.(Lys90Glufs*15)	LP (PVS1, PM2)	0	+	-	N/A	transient

										cholestasis
r-33	unknown	SLCO1B3	c.777G>A	p.(Trp259*)	LP (PVS1, PM2)	2	-	-	N/A	N/A
Prospective analysis group										
p-34	unknown	ABCB11	c.1907A>G	p.(Glu636Gly)	LP (PM2, PM3, PP1, PP3)	1	+	-	N/A	transient cholestasis
p-35	unknown	ABCB11	c.3457C>T	p.(Arg1153Cys)	LP (PS3, PM2, PP3)	7	-	-	intrahepatic cholestasis	N/A
p-36	ALGS suspected	ABCB11	c.3169C>T	p.(Arg1057*)	LP (PVS1, PM2)	0	+	+	N/A	N/A
		CFTR	c.1865G>A	p.(Gly622Asp)	LP (PS3, PM2, PP3)					
p-37	unknown	SLC25A13	c.1180+1G>A	splice-site disruption	P (PVS1, PM3, PP1)	0	-	-	N/A	N/A
p-38	unknown	SLC25A13	c.1311+1G>A	splice-site disruption	P (PVS1, PM3, PP1)	0	-	+	intrahepatic biliary hypoplasia	N/A
p-39	unknown	SLC25A13	c.1180+1G>A	splice-site disruption	P (PVS1, PM3, PP1)	1	-	-	steatosis	N/A
p-40	unknown	SLC25A13	c.852_855del	p.(Met285Profs*2)	P (PVS1, PM3, PP1)	0	-	-	N/A	N/A
p-41	unknown	ABCC2	g.(?_101590432)_(101611497_?)del	exon 21-32 deletion	LP (PVS1, PM2)	10	-	-	N/A	N/A
p-42	unknown	ABCC2	c.3928C>T	p.(Arg1310*)	P (PVS1, PM2, PM3)	0	-	+	N/A	N/A
p-43	unknown	ABCC2	c.2882A>G	p.(Lys961Arg)	LP (PM2, PM3, PP1, PP3)	0	-	-	N/A	N/A
p-44	unknown	CYP27A1	c.808C>T	p.Arg270Ter	LP (PVS1, PM2)	2	+	-	N/A	N/A
p-45	unknown	NPHP1	g.(?_110881291)_(110962603_?)del	whole exon deletion	LP (PVS1, PM2)	1	-	-	N/A	N/A
p-46	unknown	ATP8B1	c.811A>T	p.(Arg271*)	LP (PVS1, PM2)	0	-	-	N/A	N/A
p-47	unknown	CYP7B1	c.171_172insAA	p.(Glu58Lysfs*7)	LP (PVS1, PM2)	1	-	N/A	N/A	N/A
p-48	unknown	SLCO1B1	c.1683-1G>T	splice-site disruption	LP (PVS1, PM2)	0	+	-	intrahepatic cholestasis	N/A
p-49	unknown	UGT1A1	c.1005G>A	p.(Trp335*)	LP (PVS1, PM2)	0	-	-	intrahepatic cholestasis	N/A
p-50	ALGS suspected	VPS33B	c.178-1G>A	splice-site disruption	LP (PVS1, PM2)	0	+	-	N/A	N/A

ACMG, The American College of Medical Genetics and Genomics; ALGS, Alagille syndrome; BSEP, bile salt export pomp; GA, gestational age; LP, Likely Pathogenic variant; LT, liver transplantation; N/A, not applicable; P, Pathogenic variant; PFIC, progressive familial intrahepatic cholestasis; SGA, small for gestational age.

NCBI reference sequences: ABCB11, NM_003742.4; ABCB4, NM_000443.4; ABCC2, NM_000392.5; ATP7B, NM_000053.4; ATP8B1, NM_005603.6; BCS1L, NM_004328.5; CC2D2A, NM_001080522.2; CFTR, NM_000492.4; CYP27A1, NM_000784.4; CYP7B1, NM_004820.5; NPHP1, NM_000272.4; NPHP4, NM_015102.5; PEX12,

NM_000286.3; PKHD1, NM_138694.4; SLC25A13, NM_001160210.1; SLCO1B1, NM_006446.5; SLCO1B3, NM_019844.4; UGT1A1, NM_000463.3; VPS33B, NM_018668.5.

Table, Supplemental Digital Content 5. Genotypes of patients with CNS type 2

Patients	T.Bil/D.Bil, mg/dl	Allele number	Nucleotide change	Predicted amino acid change	*UGT1A1* number	UGT1A1 activity at homozygous state, % of normal
r-11	24.0/1.3	1	c.[211G>A;1456T>G]	p.[(Gly71Arg;Tyr486Asp)]	N/A	6.2
		2	c.625C>T	p.(Arg209Trp)	*UGT1A1**8	2.9
r-12	19.7/1.4	1	c.[211G>A;1456T>G]	p.[(Gly71Arg;Tyr486Asp)]	N/A	6.2
		2	c.211G>A	p.(Gly71Arg)	*UGT1A1**6	32.2
r-13	24.3/2.4	1	c.211G>A	p.(Gly71Arg)	*UGT1A1**6	32.2
		2	c.840C>A	p.(Cys280*)	*UGT1A1**25	0

T.Bil, total bilirubin; D.Bil, direct bilirubin; N/A, not applicable.

NCBI reference sequences: UGT1A1, NM_000463.3.

Table, Supplemental Digital Content 6. Genotypes of patients with Gilbert syndrome

Genotype	Allele 1	Allele 2	Number of patients		
			Retro	Pro	total
1	UGT1A1*6	UGT1A1*6	13	8	21
2	UGT1A1*6	UGT1A1*28	5	2	7
3	UGT1A1*28	UGT1A1*28	1	1	2
4	UGT1A1*28	UGT1A1*27	2	0	2

*Notation for UGT1A1 allele; UGT1A1*6 for c.211G>A, UGT1A1*28 for c.-41_-40dup, UGT1A1*27 for c.625C>T. UGT1A1 activity at homozygous state of UGT1A1*6, UGT1A1*27, or UGT1A1*6 were reported as 32.2%, 25.5%, or 14.0%, respectively.

NCBI reference sequences: UGT1A1, NM_000463.3.

Table, Supplemental Digital Content 7. The list of 150 hospitals participated in this study

Amakusa Medical Center	Kagawa University Hospital	Osaka Women's and Children's Hospital
Anjo Kosei Hospital	Kakogawa Central City Hospital	Otaru Kyokai Hospital
Asahikawa Medical University Hospital	Kanagawa Children's Medical Center	Saga University Hospital
Chiba Children's Hospital	Kansai Medical University Hospital	Saiseikai Yokohamashi Tobu Hospital
Chukyo Hospital	Keio University Hospital	Saitama Children's Medical Center
Daido Hospital	Kindai University Hospital	Saitama Medical University Hospital
Dokkyo Medical University Hospital	Kitano Hospital	Seirei Mikatahara General Hospital
Ehime Prefectural Niihama Hospital	Kobe University Hospital	Shimane University Hospital
Fujita Health University Hospital	Kumamoto City Hospital	Shizuoka Saiseikai General Hospital
Fukui Aiiku Hospital	Kumamoto University Hospital	Showa University Northern Yokohama Hospital
Fukui Prefectural Hospital	Kurashiki Central Hospital	St.Marianna University Hospital
Fukuoka Children's Hospital	Kurume University Hospital	St.Mary's Hospital
Fukuoka University Hospital	Kyoto Medical Center	Teine Keijinkai Hospital
Fukuyama Medical Center	Kyoto University Hospital	The Hospital of Hyogo College of Medicine
Gifu Prefectural General Medical Center	Kyushu Medical Center	The Jikei University Hospital
Gifu University Hospital	Kyushu University Hospital	The Jikei University Katsushika Medical Center
Gunma University Hospital	Miyagi Children's Hospital	The University of Tokyo Hospital
Hamamatsu University Hospital	Miyazaki Prefectural Miyazaki Hospital	Tohoku University Hospital
Hiroshima Prefectural Hospital	Miyoshi Municipal Hospital	Tokyo Medical University Hospital
Hiroshima University Hospital	Nagano Children's Hospital	Tokyo Metropolitan Children's Medical Center
Hokkaido Medical Center for Child Health and Rehabilitation	Nagaoka Red Cross Hospital	Tottori University Hospital
Hokkaido University Hospital	Nagoya City University Hospital	Toyama University Hospital
Hyogo Prefectural Kobe Children's Hospital	Nagoya University Hospital	Toyohashi Municipal Hospital
Hyogo Prefectural Nishinomiya Hospital	National Center for Child Health and Development	TOYOTA Memorial hospital
Ichinomiya Municipal Hospital	National Defense Medical College Hospital	Tsuchiura Kyodo General Hospital
Iizuka Hospital	Niigata City General Hospital	Tsuyama Chuo Hospital
Iwate Medical University Hospital	Niigata Prefectural Central Hospital	UJI-Tokushukai Medical Center
JA Onomichi General Hospital	Nishisaitama-chuo National Hospital	University Hospital Kyoto Prefectural University of Medicine
Japanese Red Cross Kitami Hospital	Ogaki Municipal Hospital	University of Fukui Hospital
Japanese Red Cross Medical Center	Oita University Hospital	University of Tsukuba Hospital
Japanese Red Cross Aichi Medical Center Nagoya Daiichi Hospital	Omihachiman Community Medical Center	University of Yamanashi Hospital
Japanese Red Cross Aichi Medical Center Nagoya Daini Hospital	Osaka City General Hospital	Wakayama Medical University Hospital
Japanese Red Cross Society Himeji Hospital	Osaka City University Hospital	Yamaguchi University Hospital
Jichi Medical University Hospital	Osaka General Medical Center	Yodogawa Christian Hospital
Juntendo University Hospital	Osaka University Hospital	Yokkaichi Municipal Hospital
